# Quantifying Positional Isomers (QPI) by Top-Down Mass Spectrometry

**DOI:** 10.1016/j.mcpro.2021.100070

**Published:** 2021-03-10

**Authors:** Andrea M. Brunner, Philip Lössl, Paul P. Geurink, Huib Ovaa, P. Albanese, A.F. Maarten Altelaar, Albert J.R. Heck, Richard A. Scheltema

**Affiliations:** 1 Biomolecular Mass Spectrometry and Proteomics, Bijvoet Center for Biomolecular Research and Utrecht Institute of Pharmaceutical Sciences, Utrecht University, Utrecht, the Netherlands; 2 Netherlands Proteomics Center, Utrecht University, Utrecht, the Netherlands; 3 Department of Cell and Chemical Biology, Oncode Institute, Leiden University Medical Center, Leiden, the Netherlands

**Keywords:** Top-down mass spectrometry, phosphorylation, positional isomers, ETD, Bora, ECD, electron capture dissociation, ETD, electron transfer dissociation, FDR, false discovery rate, SEM, standard error of the mean

## Abstract

Proteomics has exposed a plethora of posttranslational modifications, but demonstrating functional relevance requires new approaches. Top-down proteomics of intact proteins has the potential to fully characterize protein modifications in terms of amount, site(s), and the order in which they are deposited on the protein; information that so far has been elusive to extract by shotgun proteomics. Data acquisition and analysis of intact multimodified proteins have however been a major challenge, in particular for positional isomers that carry the same number of modifications at different sites. Solutions were previously proposed to extract this information from fragmentation spectra, but these have so far mainly been limited to peptides and have entailed a large degree of manual interpretation. Here, we apply high-resolution Orbitrap fusion top-down analyses in combination with bioinformatics approaches to attempt to characterize multiple modified proteins and quantify positional isomers. Automated covalent fragment ion type definition, detection of mass precision and accuracy, and extensive use of replicate spectra increase sequence coverage and drive down false fragment assignments from 10% to 1.5%. Such improved performance in fragment assignment is key to localize and quantify modifications from fragment spectra. The method is tested by investigating positional isomers of Ubiquitin mixed in known concentrations, which results in quantification of high ratios at very low standard errors of the mean (<5%), as well as with synthetic phosphorylated peptides. Application to multiphosphorylated Bora provides an estimation of the so far unknown stoichiometry of the known set of phosphosites and uncovers new sites from hyperphosphorylated Bora.

## Highlights

• ETD fragmentation reveals the presence of positional isomers.
• For proteins up to 40 kDa these positional isomers can accurately be quantified.
• For *in-vitro* phosphorylated BoraNT a wide array of positional isomers is revealed.
• Use of Fragment ion FDR levels improve the quality of extracted stoichiometries.

## Graphical Abstract

**Figure:**
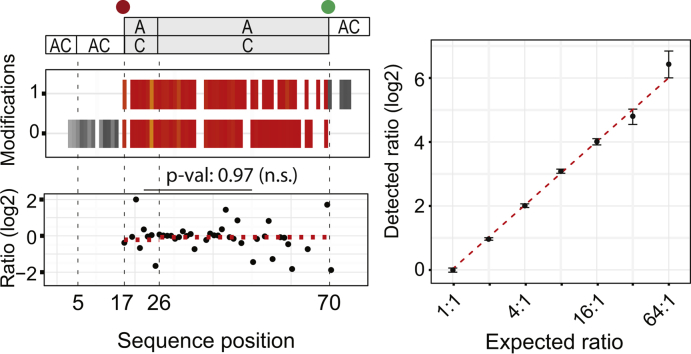

## In Brief

Here we determine the quantities of the same protein modified differentially by PTMs, creating so-called positional isomers (*i.e.*, double phosphorylation, one isomer with positions Y64 and T98, and one isomer with positions Y64 and Y120). Knowledge of the relative abundances of these separate positional isomers is highly relevant, as researchers uncovered many thousands of phosphorylation sites many of which have no clear biological function. Uncovering their relative abundance will assist in determining the relative importance of each modification site.

**Fig. 1:**
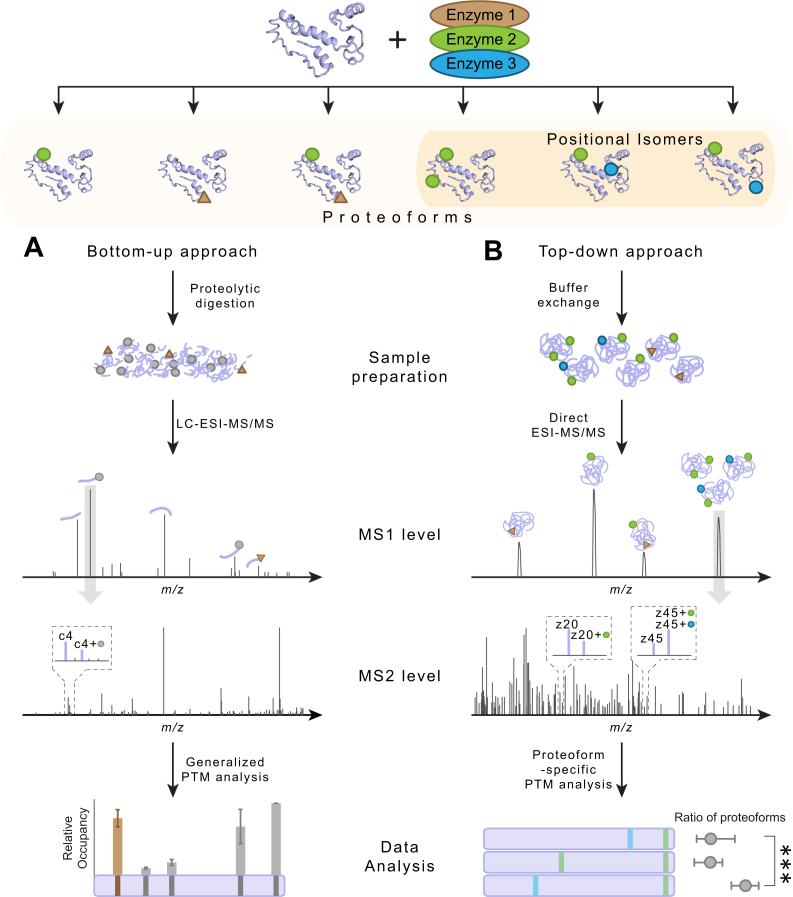
**Schematic illustration of the limitations of bottom-up MS and the benefits of top-down MS for proteoform characterization.** The defined site(s), type(s), and number of modification(s) on a protein result in a specific proteoform. Proteoforms with the same number of a certain modification type but different site(s) represent positional isomers. *A*, proteoforms cannot be characterized by traditional bottom-up proteomics, as their enzymatic digestion into peptides makes it impossible to relate peptide fragment information back to their specific proteoforms. *B*, by top-down MS of intact proteoforms, the type and number of modification(s) can be extracted from the MS1 precursor spectrum, the modification site(s), and abundance of positional isomers from the MS2 fragment spectrum.

Posttranslational modifications (PTMs) are critical in regulating protein stability, localization, activity, and interactions with other biomolecules. In the cellular environment, the number of PTMs and associated sites are often enzymatically modulated to fine-tune these activities. This creates distinct PTM configurations termed proteoforms (1, 2), potentially carrying the same number of modifications at different sites resulting in the same mass termed positional isomers (3). Dysregulation can however occur, which has been linked to inter alia neurodegenerative diseases and tumorigenesis (4, 5), creating a pressing need for methods to decipher and quantify the occurring combinations of PTMs. Bottom-up proteomics has uncovered several thousands of PTM sites, but quantification of proteoforms and positional isomers is much more difficult to achieve (6). Detection occurs at the peptide level giving a single value per modification site, which does not provide the number and co-occurrence of PTMs located on a given proteoform (Fig. 1*A*). Top-down proteomics focuses on intact proteins, ensuring that all proteoforms are measured in parallel and can be further investigated independently (7, 8, 9). The intact protein masses can be extracted from the data, from which the type and number of modifications can be inferred by the mass shifts they induce (10). In some cases, when using chromatographic separation, different peptide isomers have been demonstrated to separate in elution time and can be inspected individually to uncover the ratios between the different isomers (11). However, in cases where this is not achieved or chromatography is not employed, pinpointing the position of the modifications for a proteoform can be achieved by mass selection and fragmentation of the isoform mixture. The resulting sequence-specific fragment ions in combination with the modification-induced mass shifts indicate the PTM locations (Fig. 1*B*). While characterization of known proteoforms has been described (12, 13, 14, 15, 16), applications to discovery type experiments aimed at positional isomer characterization have been limited due to the challenges involved in separating different isomers and interpreting the acquired data (17, 18). Excellent work has already been done on developing algorithms capable of extracting the sequence identity of proteins from fragmentation spectra from full LC-MS runs, with well-described approaches to control the false discovery rates (FDRs) at the spectrum-, protein-, isoform-, and proteoform level, and additional algorithms are proposed to facilitate the identification of many different PTMs on highly modified proteins (19, 20). As these algorithms offer no FDR control at the fragment annotation level (21, 22, 23, 24), identification of positional isomers to date requires mostly manual interpretation. This has led researchers to approach the problem from an expert user-directed angle, where the presence of specific satellite ions is used to uniquely assign the different PTMs on peptides and small proteins (25).

The fragmentation scans of positional isomers (*i.e.*, proteins with the same set of modifications distributed at different locations) are highly multiplexed with specific fragments simultaneously present with and without the modification. This situation prevents automatic software to identify modification sites occluded by the modification site producing the largest number of modification carrying fragments. Even though the multiplexed spectra are hard to interpret, they also provide an opportunity to quantify the different positional isomers provided the set of positional isomers can be mass selected without interferences from other proteoforms, and the used fragmentation method does not induce structural biases in the fragment ion intensity depending on the position of the modification. This requires an ideal fragmentation technique that additionally does not incur modification losses that potentially lead to unspecific effects in the detected intensity levels of the fragment peaks, lowering the precision of the calculations or potentially even obfuscating the presence of the positional isomers. In addition, the specific modification can potentially induce unpredicted or even unpredictable effects, and modifications other than phosphorylation as investigated here should be verified in terms of accuracy. Techniques such as collision induced dissociation and higher-energy collisional dissociation readily produce such losses, and in previous studies these were also observed for ultraviolate photodissociation (26). Apart from this, the ideal fragmentation technique also leads to excellent protein sequence coverage for proteins of any size, as missing parts in the sequence coverage prevent detection of positional isomers completely. Currently, electron capture dissociation (ECD) / electron transfer dissociation (ETD) represents the best option for this set of requirements. Indeed, it has previously been reported for ECD spectra that fragment intensities correspond to their respective intact ion intensities, allowing determination of the stoichiometry of distinct histone proteoforms with known acetylation modifications (12). Additionally, it was demonstrated that ECD predominantly retains the expected intensity levels for modifications such as phosphorylation, except close to the modified site (27). In those cases where at least three amino acids are present between the positional isomers, provided those further away from the site have the correct ratio, this suggests that the ratio expressing the stoichiometry of each positional isomer can be interpreted for many positional isomers in completely unknown systems. Of note, this is only true if the indicated preconditions for ETD apply for the modification in question. To perform this task we developed a novel workflow termed “Quantifying Positional Isomers” (QPI), which utilizes a unique combination of ETD, previously reported to have minimal loss of PTMs when fragmenting peptides or proteins, and extensive bioinformatics approaches. Additionally, the technique works best with highly charged ions and full proteins provide many opportunities to carry charges. The data analysis component is combined in the application QPI, which assigns fragments and localizes modifications and uses that information to automatically calculate the stoichiometry of the positional isomers. Using Ataxin as a model system, we show that ETD fragmentation is sufficiently “soft” to leave the phosphorylation modifications in place during fragmentation, producing no losses and opening the possibility to quantify positional isomers with this fragmentation technique. With three Ubiquitin molecules differentially modified in distinct positions and mixed in well-defined ratios, we show that stoichiometries can be obtained from fragmentation spectra for nonlabile modifications representing the minimal case where the method should work. Of note, modifications carrying charges or of large size can have influence on the ratios; *e.g.*, it was demonstrated that the presence of a phosphorylation site can have a suppressing effect on the fragmentation intensity of the fragment containing this site (27). We go on to show that ETD on phosphorylated peptides produces the expected ratio from the fragmentation spectra when mixed in predefined ratios for five amino acids (or fragment intensities) between the positional isomers; we anticipate distortions when this number drops below 5, and it becomes impossible to calculate a correct ratio when the number drops below 3. Finally, we apply the approach to uncover the positional isomers of *in vitro* phosphorylated Bora (a mitotic regulator) and detect their relative abundances, for which we go up to three phosphorylation sites. Incredibly, we can detect for doubly phosphorylated Bora six distinct positional isomers and provide estimations for the quantities for each. For triply phosphorylated Bora the situation is even more complex, as we find 13 distinct positional isomers. As it currently stands, the technique still has limitations with the complexity introduced with such numbers of isomers, and we are not (yet) able to extract estimations of all individual abundances. It is evident that the current generation of deisotoping procedures introduce additional variation at the level of incorrectly assigned monoisotopic masses, splitting peaks, and other problems leading to incorrect intensity values. We deal with the aforementioned problems by introducing grouping of different positional isomers to a single ratio. Even though from this the exact contributions of each positional isomer cannot be extracted, we can narrow the highest abundant positional isomer as one with two positions with high confidence combined with one of four positions. Such knowledge can help to direct and specifically limit mutation studies to relatively few positions to help determine the biological relevant combination of sites.

## Experimental Procedures

### Chemicals and Reagents

Formic acid (FA) was purchased from Merck; acetonitrile (ACN) and methanol (MeOH) were purchased from Biosolve. The catalytic subunit of cAMP-dependent Protein Kinase (PKA) used to *in vitro* phosphorylate proteins was purchased from NEB (Ipswich). We utilize three different, combinatorially expressed proteins that were previously characterized in detail. Ataxin^562–815^ was provided by Prof. Luc Brunsveld (Technische Universiteit Eindhoven) and was prepared as previously described (28). Heavy labeled ubiquitin proteoforms were synthesized by solid-phase peptide synthesis according to the reported procedure (29). Fmoc-labeled heavy valine (^13^C_5_, ^15^N) (Cortecnet) was introduced at the appropriate positions. And finally, for Bora we utilized the recorded data as presented in Lössl *et al.* (3).

### Kinase Reactions

Ataxin^562–815^ was phosphorylated through incubation with PKAc in 50 mM Tris-HCl, 10 mM MgCl2, 1 mM ATP pH7 for 1 h at 30 °C. Reactions were quenched by addition of 20 mM EDTA pH 8.0. Prior to top-down MS analysis, phosphorylated proteins were denatured by buffer exchange to 0.1% FA using 5 kDa molecular weight cutoff centrifugal filter units with polyethersulfone membranes (Sartorius).

### Mass Spectrometry

All data was acquired on a Thermo Scientific Orbitrap Fusion mass spectrometer (Thermo Fisher Scientific) in direct infusion experiments. Ataxin^562–815^ was diluted to a final concentration of 5 μM in 50% ACN, 1% FA, and ubiquitin to 2.5 μM in 50% MeOH, 1% FA. Protein samples were sprayed at a flow rate of 1 μl/min. Data were acquired in the Orbitrap mass analyzer at a resolution of 120,000 for Ataxin^562–815^ and 240,000 for ubiquitin (full width at half-maximum, FWHM) in intact protein mode (2 mTorr ion-routing multipole (IRM) pressure). The presence and number of modification(s) were determined based on the monoisotopic mass of the isotopes detected in the MS1 spectra. Of each sample and each setting change, a set of triplicate measurements was recorded; each replicate was recorded as follows. Specific proteoforms were isolated with the mass selecting quadrupole and an isolation width of 1 Th for Ataxin^562–815^ and 2 Th for ubiquitin. We selected the more narrow isolation window 1 Th for Ataxin to prevent any coisolation of peaks in close proximity due to the elevated charge states, which represent a different phosphorylation state. The 37+ charge state of intact, nonphosphorylated Ataxin^562–815^ at 748Th and the 38+ charge state of doubly phosphorylated Ataxin^562–815^ at 733 Th were subjected to ETD fragmentation with 2, 4, and 6 ms ion/ion reaction time. The 11+ charge state of ubiquitin at 778 Th was subjected to ETD fragmentation with 6 ms reaction time. MS2 spectra were acquired using a mass range of 150 to 2000 m/z. A total of 500 microscans were summed for Ataxin^562–815^ and 100 for ubiquitin spectra.

### Data Analysis

Deconvolution of intact protein and top-down spectra was performed in Protein Deconvolution 4.0 (Thermo Scientific) using XTRACT with a signal-to-noise ratio (S/N) threshold of 1.1, a fit factor of 40%, and a remainder threshold of 15%. Deconvoluted spectra were analyzed with QPI, a software we developed in-house for proteoform characterization written in C#. It takes as input (i) the protein sequence and (ii) the deconvoluted top-down spectra. Through the described procedures the fragment ions are assigned and identified at FDR rates <1%. Assignments are organized in PTM ladders, which are constructed by progressively adding the delta mass of the desired modification to the fragment masses. Visualization in a heatmap format allows the operator to inspect and manually pinpoint the modification sites. After calculating the ratios between n and n + 1 modifications in the heatmap, a simple *t*-test can be applied to determine whether a positional isomer exists between two possible locales for the PTM under investigation.

## Results and Discussion

### Improving the Confidence of Fragment Assignment in Top-Down Mass Spectra

**Fig. 2:**
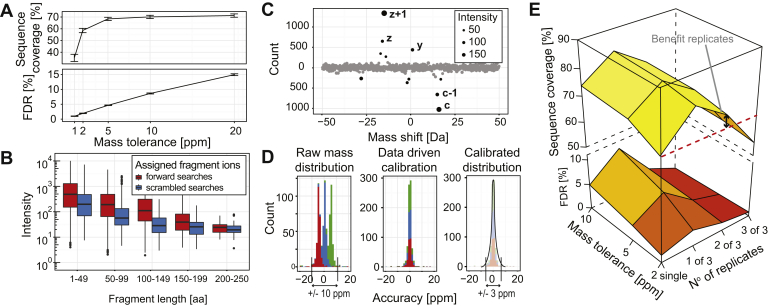
**Data curation and fragment assignment in QPI results in low false-positive assignments and high protein sequence coverage.***A*, effect of mass tolerance as a parameter on the FDR rate. *B*, intensity (in-)dependence of forward and scrambled searches. *C*, frequent flyer fragment-type definition automatically extracts which fragment ions to consider in fragment assignments. *D*, the raw mass distribution of a data set generated by ETD top-down MS of intact Ataxin^562–815^ is shown in *blue*, entirely falsified spectra created by adding and subtracting, respectively, 5 Da to/from every monoisotopic peak in every real spectrum in *green* and *red* (*left panel*). Data-driven mass calibration corrects the median value by the systematic mass deviation (*mid panel*) and reduces the accepted mass tolerance to <3 ppm (*right panel*). *E*, extensive data curation and majority voting for combination of multiple spectra result in FDRs <1% and the replicates provide the benefit of higher sequence coverage.

Confident PTM localization is ideally based on protein sequencing data with no false fragment assignments and high sequence coverage. This requirement is needed as, in extreme cases, incorrectly assigned fragments potentially obfuscate the correct position leading to an incorrect assignment. Furthermore, missing fragments can make it impossible to pinpoint the site when multiple acceptor residues are closely spaced in the sequence. False positives however cannot be readily excluded and, to our knowledge, FDR calculation at the fragment ion level was not addressed in standard top-down MS software. To benchmark the FDR of fragment ion assignments in top-down fragmentation spectra we used the well-characterized AXH domain of human Ataxin-1 (Ataxin^562–815^) and searched its fragment spectra against 200 different scrambled sequences to calculate the percentage of the sequence that can randomly be explained (defined as the median sequence coverage of the 200 searches). This can readily be automated with standard string randomization approaches accessible in any programming language. By matching the theoretical fragments from these randomized sequences to the deconvoluted spectra, the sequence coverage can be calculated. The fragment level FDR is then defined as the percentage of the full sequence length that can on average be covered by 200 randomized sequences. Based on this FDR metric, we find that using standard parameters (*i.e.*, setting mass tolerance to 10 ppm, combining multiple spectra, and allowing fragment ions a, b, c, x, y, and z with neutral losses water, ammonia, and hydrogen activated) results in an unacceptably high FDR of 9% at 70% sequence coverage for unmodified Ataxin^562–815^, partially resolvable by using a more stringent mass cutoff for fragment matching (Fig. 2*A*). For optimization of the FDR, we initially sought to use a fragment ion intensity cutoff; however, searches against scrambled databases revealed no substantial correlation between false-positive assignments and intensity (Fig. 2*B*).

Exclusively using the fragment ion types actually present in the fragmentation spectrum is a more effective measure. To determine the relevant ion types, we utilized a “frequent flyer” algorithm, which determines those ion types actually encountered in each individual spectrum (30). This strategy works well for top-down fragmentation spectra as each spectrum contains thousands of annotatable fragment ions. For instance, ETD fragmentation of Ataxin^562–815^, which at ∼27 kDa is a mid-sized protein in top-down analyses, generated ∼2000 fragment ions. The ion types c, c − 1, y, z, and z + 1 were found to be significantly present based on a significance A test (31) as the fragment ion types of interest (Fig. 2*C*). This is in line with previous reports of peptide ETD MS that showed that c, y, and z ions were predominantly generated by ETD fragmentation (32). Re-searching the Ataxin^562–815^ data allowing only for the defined ion types c, c − 1, y, z, and z + 1 reduces the FDR to 4% for the same sequence coverage. A further reduction of the search space, and thus the FDR, is achieved by higher stringency on the mass error tolerance used for fragment ion matching. This however requires mass calibration of the recorded mass spectra to remove systematic errors introduced during data acquisition. Analogous to previously reported post data acquisition approach for mass calibration (33, 34), QPI calculates the mass deviations from the entirety of annotatable fragment ions. This results in a normally distributed population demonstrating no m/z range dependency, and any systematic mass deviation within the population can be removed by correcting the median value by this shift (supplemental Fig. S1). Additionally, the normally distributed full population of mass deviations allows for the calculation of the allowed mass tolerance, which we define as 3x standard deviation (Fig. 2*D*). Generally this results in an acceptable mass precision of <3 ppm, providing a very stringent filter for fragment ion assignment, which further reduces the FDR to ∼2% for a decreased sequence coverage of 62%. Finally, we hypothesized that false-positive assignments of fragment ions can be reduced by considering technical replicate scans. Accordingly, a true-positive fragment should be found in more replicate scans than false-positive random matches, which can be exposed by a “majority voting” algorithm filtering the annotations of all technical replicates based on a minimum occurrence rate. We find that combining replicate ETD scans, *i.e.*, subsequent scans from a single run, increases protein sequence coverage in a similar manner as reported previously for combining scans of multiple different fragmentation methods (18). However, we find here that this practice also increases the FDR, potentially impeding unambiguous modification site localization. Indeed, starting with a ∼2% FDR and 65% sequence coverage for single ETD fragmentation scans, the FDR initially almost doubled to ∼4% when fragment ions of three replicate scans were combined. Only considering fragment ions that occur in at least two out three technical replicates decreased the FDR rapidly to ∼1% and still improved the sequence coverage as compared with single scans from 65% to 70%. Filtering for fragment ions that are present in all replicates further reduced the FDR to <1% but also caused a substantial drop in sequence coverage to 55% (Fig. 2*E*). Therefore, we chose to accept fragment ions present in at least two out of three replicates, as this represents the best compromise between boosting sequence coverage and reducing FDR.

The improved sequence coverage at low false-positive rates of this compromise improves the likelihood of correctly localizing PTMs as misassignments are very rare and holes in the sequence coverage will not complicate localization. As such, automated approaches are expected to generate more reliable results.

### Using PTM Ladders for Site Localization

**Fig. 3:**
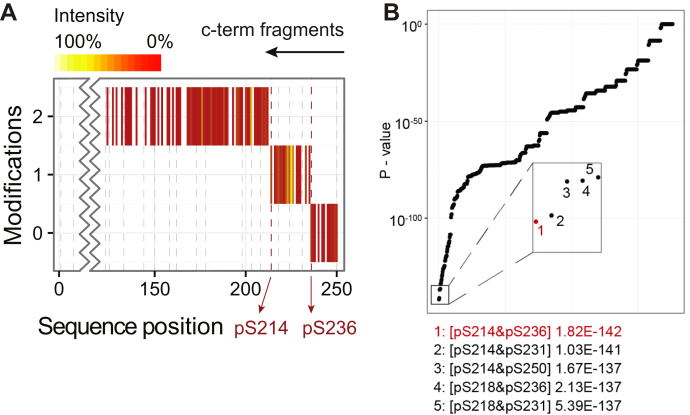
**PTM localization with sequence ladders.***A*, PTM ladders localize modification sites from the fragmentation spectra. *B*, PTM localization probabilities for doubly phosphorylate Ataxin.

In contrast to other approaches implementing automatic modification site assignment (21, 23, 35), in QPI, an increasing number of modifications are added to both the N and C termini of the input protein sequence. This leads to PTM ladders for the N and C termini individually, defined as sequence stretches covered by fragment ions with no modification, followed by stretches covered by ions with one modification, with two modifications, and so forth up to the maximum number of possible modifications determined from the precursor mass. The resulting modification ladder shows at which amino acid residue(s) a new modification occurs (see supplemental Fig. S2). Demonstrating this procedure for doubly phosphorylated Ataxin^562–815^, where the modification ladders show no signs of modification loss during ETD fragmentation, both phosphorylation sites can be unambiguously assigned to Ser214 and Ser236 (Fig. 3*A*).

Automatic site localization however requires a likelihood score to separate the correct site from all possibilities, which can result into many thousands of combinations. To illustrate, for our simpler system of doubly phosphorylated Ataxin^562–815^ (considering that S, T, and Y can be phosphorylated) a total of 703 unique combinations of phosphorylatable amino acids can be made. To implement such a score we utilize a probability calculation established for peptide fragmentation spectra (36) and modify it for the PTM ladders generated by top-down fragmentation spectra. The adaptation consists of application of the score to each ladder—or stretch between modifiable residues, resulting in stretches approximately the length of the peptides the score originally was developed for—and finally combination of all the individually calculated scores. The basis for this calculation is a probability for a theoretical fragment to match by chance, which we approximate by calculating the average number of peaks (X) per 100 Da in the spectrum, as opposed to using the number from the TopX filtering. The theoretical chance then equates to “X/100” as previously described. To determine the probability of a particular combination of modification sites, we calculate the match probability for each stretch at the number of modifications based on the number of matched and theoretical peaks for that stretch. For example, in the case of the combination of 214 and 236, we calculate the probabilities: for 0 modifications, residues 0 to 214; for one modifications, residues 215 to 236; and for two modifications, residues 237 to 255. All these probabilities are multiplied to get to the final result. This however ignores small stretches “in-between” a main stretch. For example, instead of residue 236 the residue 231 can also be modified. As the difference is relatively small and potentially underrepresented with matches it can happen that this skews the end score to favor the shorter stretch. To resolve this, we correct each individual *p*-value by dividing it by the match probability *p*-value of the previous stretch prior to multiplication. Application to Ataxin^562–815^ indeed finds the best *p*-value for the combination serine 214 and serine 236. This combination scores 1.8e-142 and is followed by 214 + 231 at 1.0e-141, clearly separating the two, and thereby the correct position pair is selected (Fig. 3*B*).

### Quantifying Positional Isomers

The ultimate goal is the quantification of positional isomers. They are visible in the PTM ladders as sequence positions carrying different numbers of modifications and their relative ratios potentially reveal the stoichiometry of each of the positional isomers. To demonstrate this, we used synthetic ubiquitin with a heavy isotope-labeled Valine positioned at residue 17, 26, or 70 (A, B, and C respectively) as a model system. Measuring these positional isomers independently results in the anticipated sequence ladders for positional isomers A and C individually and localizes the modifications at the second modifiable position for protein A (Val17) and the fourth modifiable position for protein C (Val70) (supplemental Fig. S3). To verify that the position of the heavy labeled Valine does not result in differences in the intensity levels at their location point in the sequence, we investigated the fragmentation spectra. From the mirror plot, it is evident that the overall fragmentation behavior is highly similar for both positions even though these are two separate fragmentation scans (supplemental Fig. S4*A*). After matching the peaks for the same sequence position, we observe an excellent correlation coefficient of 0.97 between the detected fragment intensities (supplemental Fig. S4*B*). To verify whether this holds for the combination of ETD and phosphorylation, we tested the quantitation on synthesized phosphopeptides. From the mixing ratio and the detected ratio from the fragment spectra, we conclude that the fragment ion intensities are not significantly affected by either position or phosphorylated residue (supplemental Fig. S5).

**Fig. 4:**
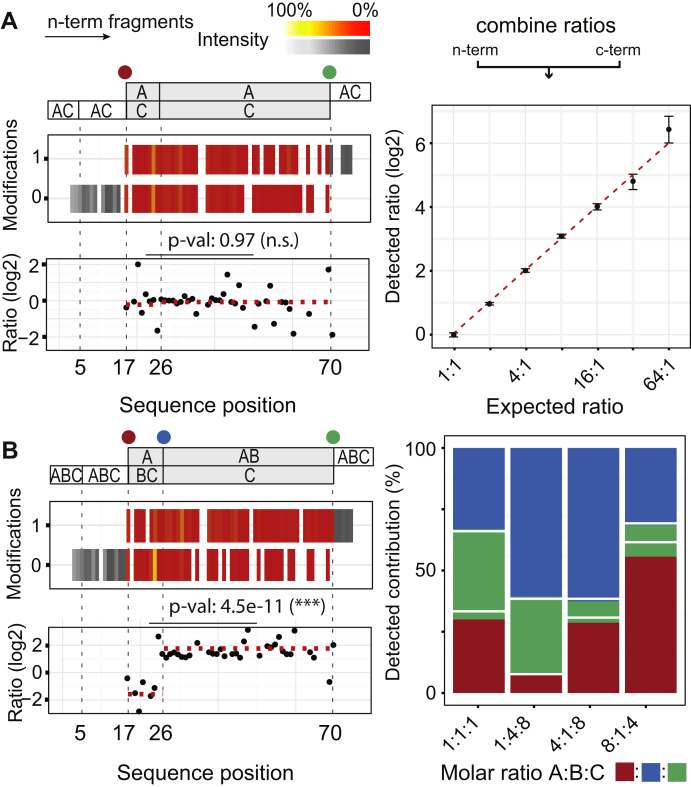
**Quantification of positional isomers in the Ubiquitin model system.***A*, for positional isomers modified and unmodified fragments are observed in the PTM ladders for individual sequence positions (*top-left*), the ratio of which is reflective of the mixing ratio (in this case 1-to-1; *bottom-left*). Performance on a range dilution of mixing ratios shows correct ratios up to extreme levels (*right*). *B*, introduction of an extra positional isomer causes higher complexity (*top-left*) and shifts in the ratios that can be detected by Student’s *t*-test (*bottom-left*). Performance of various mixing ratios shows correct values also for more complex positional isomer arrangements (*right*; *white line* is the expected value).

Mixing positional isomer A and C in a 1:1 ratio in our Ubiquitin model system results in modified and unmodified fragments in the sequence stretches 17 to 26 and 26 to 70, with the N-terminal modified fragments belonging to isomer A and the unmodified to isomer C (Fig. 4*A*; *top-left panel*). Their respective fragment intensity values are indicative of their amount and were used to calculate a ratio expressing the stoichiometry of the positional isomers (Fig. 4*A*; *bottom-left panel*). Considering both N- and C-terminal fragment ions separately adds a level of confidence to the ratio determination. The median ratios of the N-terminal fragment ion pairs (eight out of nine for stretch 17–25 and 32 out of 44 for stretch 26–69) and the C-terminal fragment ion pairs (seven out of nine for stretch 18–26 and 35 out of 44 for stretch 27–70) all result in a value of 1 (standard error of the mean (SEM) < 0.05%), reflecting the expected 1:1 mixing ratio. Extending the mixing ratios of the isomers up to 64:1 results in an experimental calibration curve matching the theoretically expected quantitative results, exhibiting stable SEM values (∼2%) up to a ratio of 32:1 (Fig. 4*A*; *right panel*). For the 64:1 mix, ratio compression starts to occur, a phenomenon previously described for MS-based relative quantitation of peptides (37, 38), and the detected fragment abundances of the low concentration positional isomer become less reliable.

In many cases, positional isomers will involve other residues than the first and last modified residue of the modification ladder, *e.g.*, a heavy labeled Val26 in addition to the modified Val17 and Val70 in case of ubiquitin. The presence of additional isomers cannot be determined based on the maximal number of assigned fragments. However, as their presence will affect the ratio of modified and unmodified fragments in the individual stretches between modified sites, this ratio can be used to identify whether positional isomers are present. To achieve this, a *t*-test compares the fragment ion ratios of all potentially modified sites. A statistically significant difference (*p*-value < 0.05) between the fragment ion ratios of individual sequence stretches is indicative for an additional modification site, hence the presence of additional positional isomers. In the case of the 1:1 mixture of positional isomers A and C, we find a *p*-value of 0.97 and conclude that Val26 is not modified, and no further isomers are present (Fig. 4*A*). To illustrate the reverse, we added positional isomer B (modified at Val26) to the ubiquitin protein mixture. The resulting situation is visualized for N-terminal fragments, showing that the fragment ion complexity has substantially increased (Fig. 4*B*; *top-left panel*). Modified fragments in the sequence stretch 17 to 25 belong to isomer A and unmodified fragments to isomers B and C, whereas modified fragments in the sequence stretch 26 to 69 belong to isomers A and B and unmodified fragments to isomer C. The contribution of A, B, and C can thus again be extracted from the fragment ion intensities. When considering a 1:1:1 mixture the detected ratios display, as expected, different behavior over the stretches 17 to 26 and 26 to 70. Comparing both stretches using the t-test results in a *p*-value of 4.5e-11, showing that their ratios are significantly different and therefore Val26 is modified (Fig. 4*B*; *bottom-left panel*). In our data with triple mixing ratios (1:1:1, 1:4:8, 4:1:8, and 8:1:4), this approach retrieves all the expected ratios (Fig. 4*B*; *right panel*). It should be noted that this procedure is not limited to two or three positional isomers. Rather, every additional positional isomer gives rise to a new unknown variable, for which a new stretch is introduced. The ability to solve this mathematical problem thus mainly depends on a sufficiently differing ratio and on the number of provided input parameters and the quality of the experimental data (see supplemental Fig. S6).

### Application to Phosphorylated Bora

**Fig. 5:**
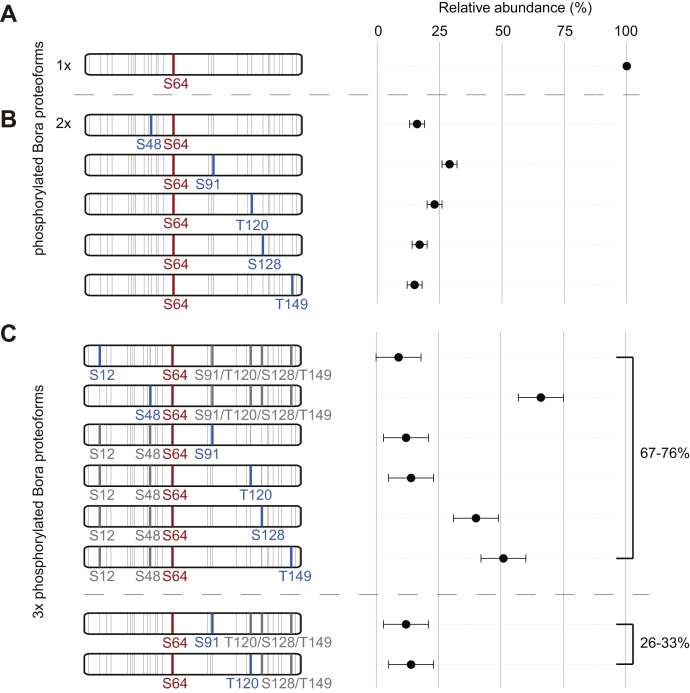
**Quantification of positional phospho-isomers of Bora**^**NT**^**.***A*, in the singly modified protein, one proteoform is present with a phosphorylation site at S64. *B*, in the doubly modified protein, five additional sites are phosphorylated in combination with S64. All positional isomers are present in roughly similar relative abundances. *C*, in the triply phosphorylated protein, the exact relative abundance of the individual positional isomers cannot be resolved; however, grouped quantification allows extraction of a maximum relative abundance of each proteoform. Here 67 to 75% are proteoforms comprising pS64 and either pS12 or pS48 and a C-terminal modification (*bold gray lines*); and 26 to 33% are proteoforms comprising pS64 and either pS91 or pT120 and a C-terminal modification.

Next, we reinvestigated data from the 17.5 kDa N-terminal domain of the mitotic regulator Bora (BoraNT), which we reported before (3). As shown previously, BoraNT can be phosphorylated *in vitro* by Aurora kinase A, resulting in a variety of positional isomers starting with its doubly phosphorylated proteoform (18). With 30 phosphorylatable residues in the 156 amino acid sequence, some in close proximity, proteoform assignment is a difficult task, which we attempt to resolve with our approach. The site of the singly phosphorylated proteoform was correctly localized to Ser64 (see Fig. 5*A* and supplemental Fig. S7). As the PTM ladder shows a single step, without exhibiting overlap of non-modified and singly modified fragments, we conclude 100% occupancy for this phosphorylation site. The doubly phosphorylated proteoform revealed 100% occupancy of Ser64, but the second phosphate moiety is distributed over five additional sites (Ser48, Ser91, Thr120, Ser128, and Thr149). QPI resolved and quantified these five distinct positional isomers with relative abundances ranging from 15% to 29% (see Fig. 5*B* and supplemental Fig. S8). From the overlapping no, singly, and doubly phosphorylation carrying fragments, we conclude the indicated six modified sites (based on the procedure described above). The combination of these results in 15 theoretically possible proteoforms representing 15 unknowns. The seven data points achieved experimentally do not allow proteoform quantification; however, from the singly phosphorylated Bora it is evident that S64 has 100% occupancy. Applying this constraint reduces the number of unknowns to five potential proteoforms, which can be quantified with the seven experimentally determined data points. Tackling the next level of complexity, 3x phosphorylated BoraNT, an additional site was observed at Ser12, resulting into 13 distinct positional isomers for the triply phosphorylated BoraNT (see Fig. 5*C* and supplemental Fig. S9). The combination of these results in 35 theoretically possible proteoforms, *i.e.*, 35 unknowns. The seven data points achieved experimentally do not allow proteoform quantification. As before, the data show that S64 is always phosphorylated and phosphorylation at S12 and S48 and S128 and T149 are mutually exclusive. The exclusivity between S12 and S48 arises from the requirement that S64 is always present and only fragments with three modifications after S48 are detected in the set of C-terminal fragments. Similarly, exclusivity between S149 and S128 arises from the requirement that S64 is always present and only fragments with one modification up to S120 are detected (*i.e.*, either S149 or S128 is present). Applying these constraints leads to 13 potential proteoforms that cannot be quantified with the seven experimentally determined data points, although they could be quantified if all 14 theoretically possible data points had been achieved. However, grouping positional isomers that cannot be disentangled indicates the maximum relative abundance of the specific proteoforms (Fig. 5*C*). The SEM of 10% indicates that for this system, less accurate estimations can be made for this sample, which also exceeds our ability to quantify all positional isomers.

## Discussion

The combination of PTMs and their cross talk are critical in cellular processes. To fully live up to its potential, proteomics needs to decipher which combinations of modifications occur on a protein in a defined condition. This would allow researchers to elucidate how physiological and pathological states impact the dynamic regulation of PTMs and the cross talk among PTM sites. QPI attempts to address precisely this need with its ability to identify proteoforms, including positional isomers, and—within the theoretical limitations—quantify them. The proof-of-concept applications to isotopically labeled ubiquitin and (hyper)phosphorylated BoraNT demonstrate that the QPI approach can provide estimations of the quantities of positional isomers, but also reveal the intrinsic complexity due to the exploding numbers of co-occurring isoforms when the number of attached PTMs increases. In addition, it is worth noting that some PTMs such as phosphorylation can distort the fragment intensities close to the modification site. Even though our approach is expected to be robust in those cases where at least five amino acids provide fragment intensities next to the modification site due to the use of the median, the ratios with SEMs exceeding 10% (determined from the high noise, triply phosphorylated Bora experiment presented here) should be treated with caution. This also means that the method is not generally applicable, *e.g.*, in cases where the number of amino acids or fragment intensities becomes too low to calculate a reliable ratio. In cases where the ratio cannot be calculated or where the SEM is excessively large, orthogonal methods are required to calculate the correct ratios. Further investigations into advancing the investigation into positional isomers are therefore required as, as far as we are aware, no methods exist to date.

The theoretical limits of the approach apply to how much data can be extracted from the spectrum and how many “unknowns” are present as positional isomers. When the number of unknowns exceeds the number of data points that can be extracted from the spectrum, the calculation cannot be resolved and additional data must be collected. We envision that this is possible with higher-resolution separation methods able to disentangle positional isomers and/or with MS instrument improvements such as on-the-fly targeted MS3 sequencing steps of internal fragments, which together may solve the quantification of proteoforms that exceed the current decipherable number of PTM sites and combinations. To partly deal with the current level of data quality, we introduced grouped quantitation where for a group of positional isomers the stoichiometry can be extracted. How each individual positional isomer behaves will need to be disentangled by other methods, but the required work is severely limited to a small set of positional isomers. Another limitation of the approach is that only a single modification can be taken into account. As for multiple modifications, the theoretical limits are even more readily achieved. There are software solutions available that can handle multiple modifications, but restrict there on the polypeptide sequence length. As QPI is designed to deal with full proteins, this currently is not feasible.

## Data Availability

The mass spectrometry proteomics data have been deposited to the ProteomeXchange Consortium (http://proteomecentral.proteomexchange.org) *via* the PRIDE partner repository with the data set identifier PXD012084. Furthermore, the software is available in the supplementary material as zip file containing the executables, a manual, and an example data set.

## Supplemental data

This article contains supplemental data (39)

## Conflict of interest

The authors declare no competing interests.
