# Supplementary material for: Quantifying Positional Isomers (QPI) by Top-Down Mass Spectrometry
Source: Mol Cell Proteomics. 2021 Mar 10;20:100070. doi: 10.1016/j.mcpro.2021.100070 (PMC8099777; doi:10.1016/j.mcpro.2021.100070)
Supplement: Supplemental Figures S1–S9 [file mmc1.docx]

**Supplementary Material**

**Quantifying Positional Isomers (QPI) by top-down mass spectrometry**

Andrea M. Brunner^1,2^; Philip Lössl^1,2^; Paul P. Geurink^3^, Huib Ovaa^3,$^, P. Albanese^1,2^, A.F. Maarten Altelaar^1,2^; Albert J. R. Heck^1,2^; Richard A. Scheltema^1,2,^*

1. Biomolecular Mass Spectrometry and Proteomics, Bijvoet Center for Biomolecular Research and Utrecht Institute of Pharmaceutical Sciences, Padualaan 8, 3584 CH Utrecht, Utrecht University, The Netherlands
2. Netherlands Proteomics Center, Utrecht University, Utrecht, The Netherlands
3. Department of cell and chemical biology, Oncode Institute. Leiden University Medical Center. Leiden, the Netherlands.

*corresponding author: [r.a.scheltema@uu.nl](mailto:r.a.scheltema@uu.nl)

$ deceased May 19, 2020


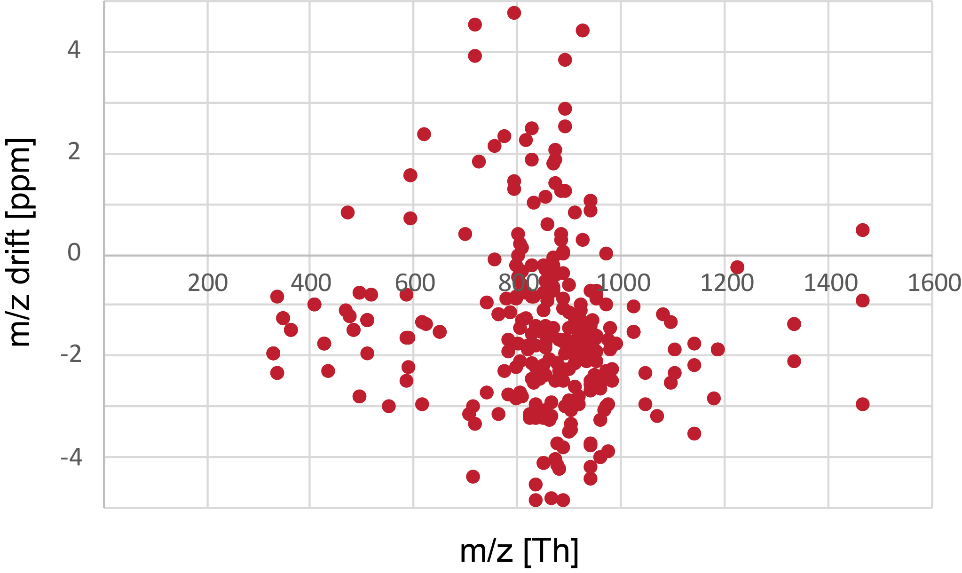


**Supplementary Figure 1 –** No m/z effects on mass deviation.

**
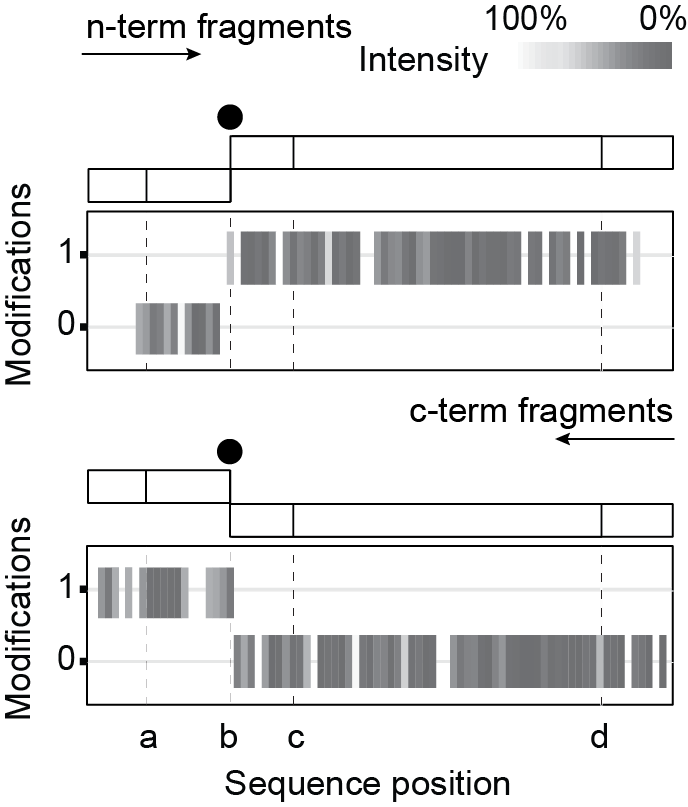
**

**Supplementary Figure 2 – Modification ladders localize modification sites.** Exemplary sequence stretches covered by fragments without and with 1 modification, respectively, form a modification ladder. For this fictive protein with the potential PTM sites a, b, c and d, the PTM site can be assigned to b, as the fragment ions carrying 1 modification start at position b.

**
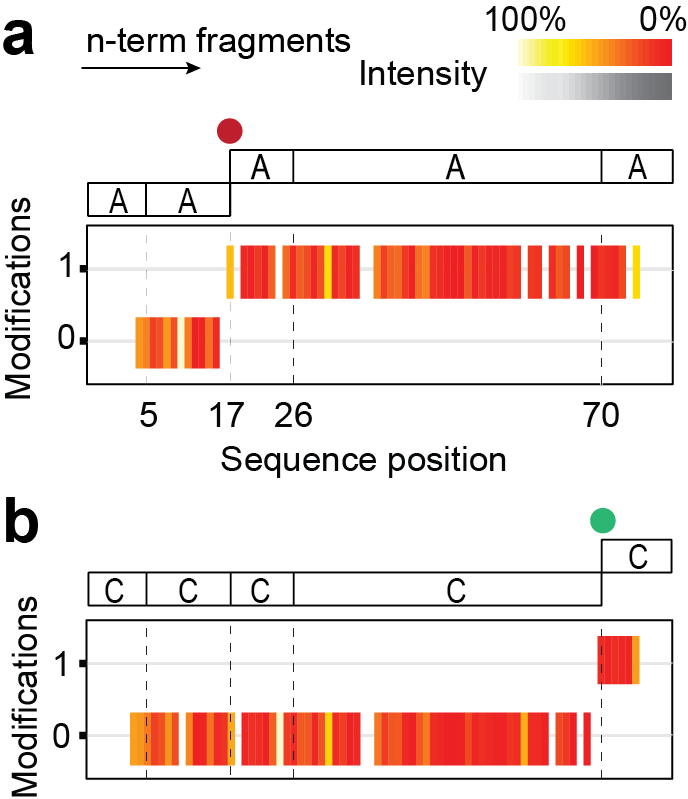
**

**Supplementary Figure 3 – Sequence ladders of the ubiquitin gold standard model system of positional isomers.** An ubiquitin model system was synthesized with heavy Valine labels at positions Val 17 (A, depicted in red), Val 26 (B, not shown), and Val 70 (C, green) for use as a proxy for positional isomers. Schematic depiction of positional isomers A (a) and C (b), and their respective heatmaps showing modified and unmodified fragment intensities. The modification ladders show that the labeled residue is Val17 in positional isomer A, and Val70 in positional isomer B.

**
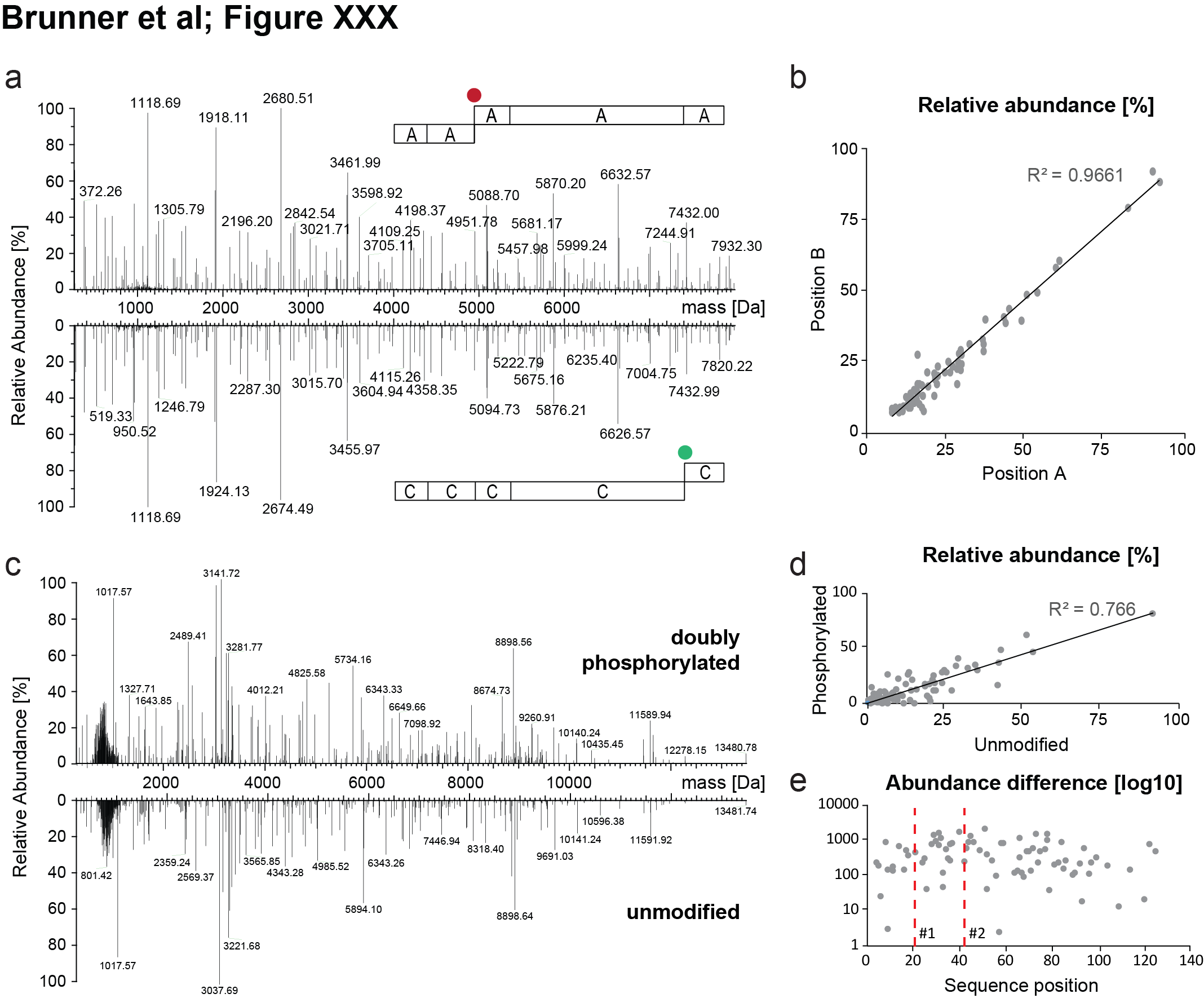
**

**Supplementary Figure 4 – Comparison of fragment intensities in separately recorded spectra. (a)** Mirror plot of the deconvoluted spectra of heavy labelled Ubiquitin modified in different locations (precursor removed); position A (top) and position C (bottom). **(b)** Correlation plot of the detected fragment intensities.


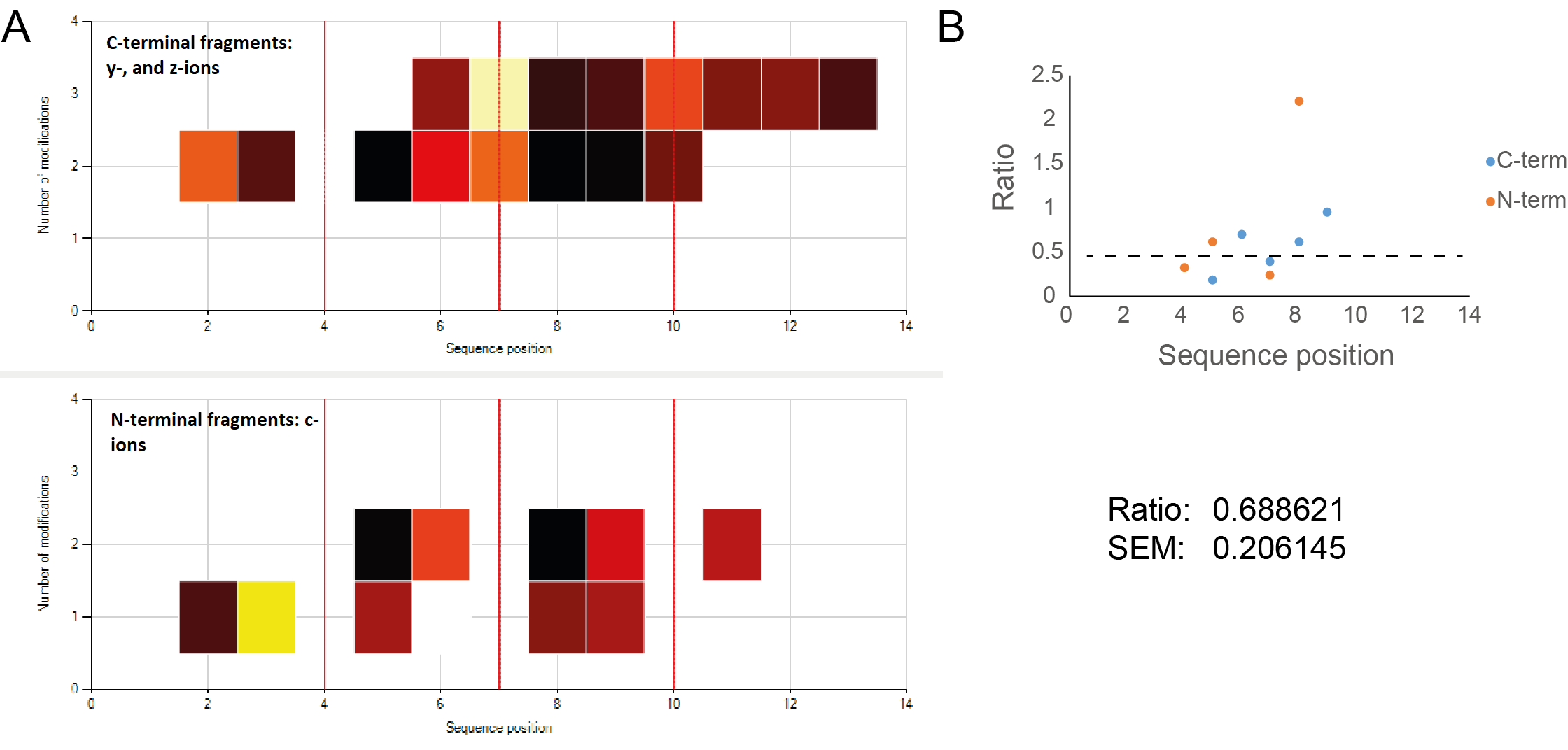


**Supplementary Figure 5 – ETD fragmentation of synthetic peptide FIGSPRTPVSPVK phosphorylated at either S(4) or S(10) mixed in a 2:1 ratio.** The peptides were synthesized and quantified as described by Schmidlin *et al*(1) and annotated with the fragmentation rules described in the manuscript. **(A)** Heatmap representation of the extracted fragment intensities (colors are heat-coded, running from black to red to yellow/white). **(B)** Extracted ratios by QPI.


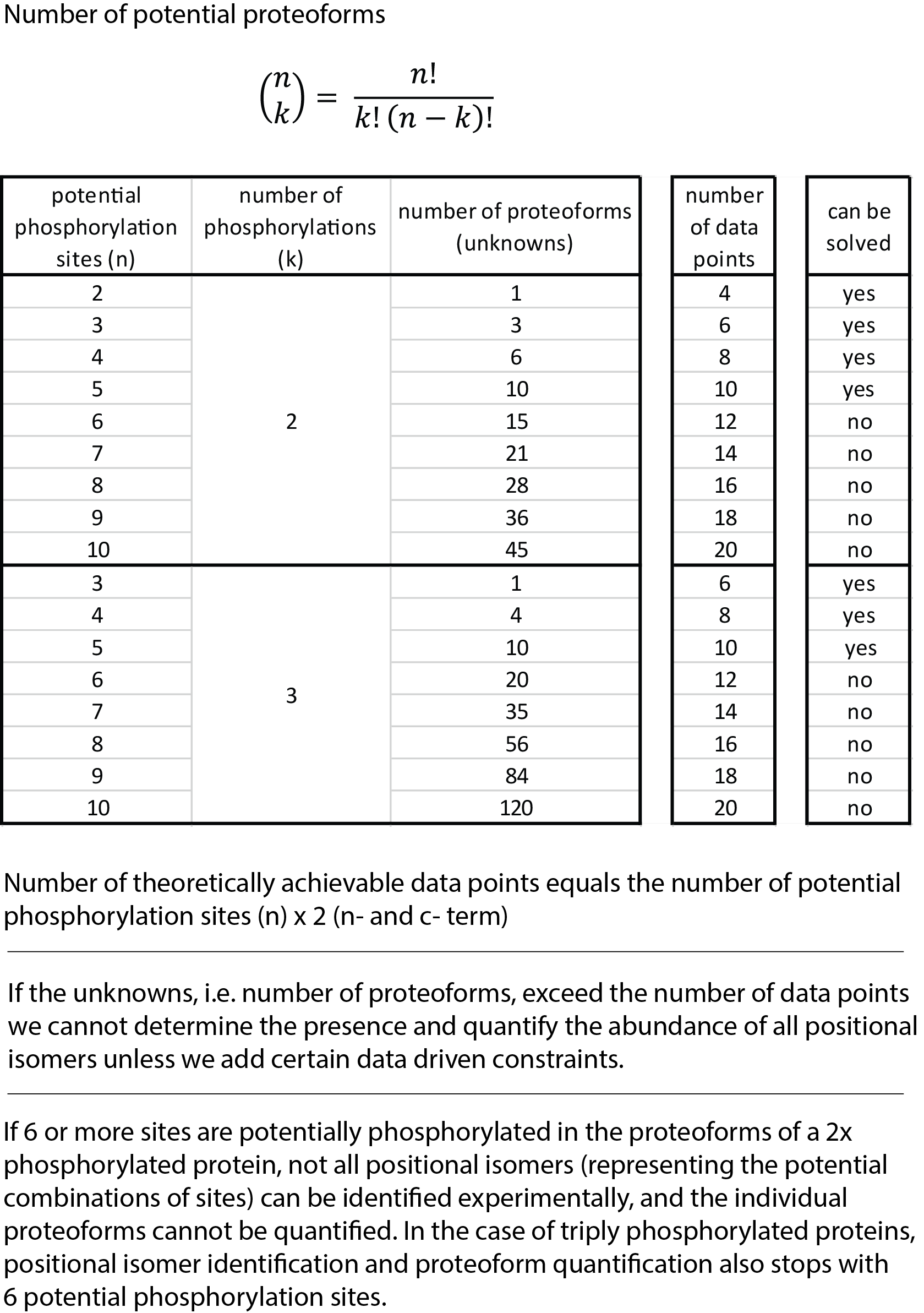


**Supplementary Figure 6 – Theoretical limitations to positional isomer quantification.**


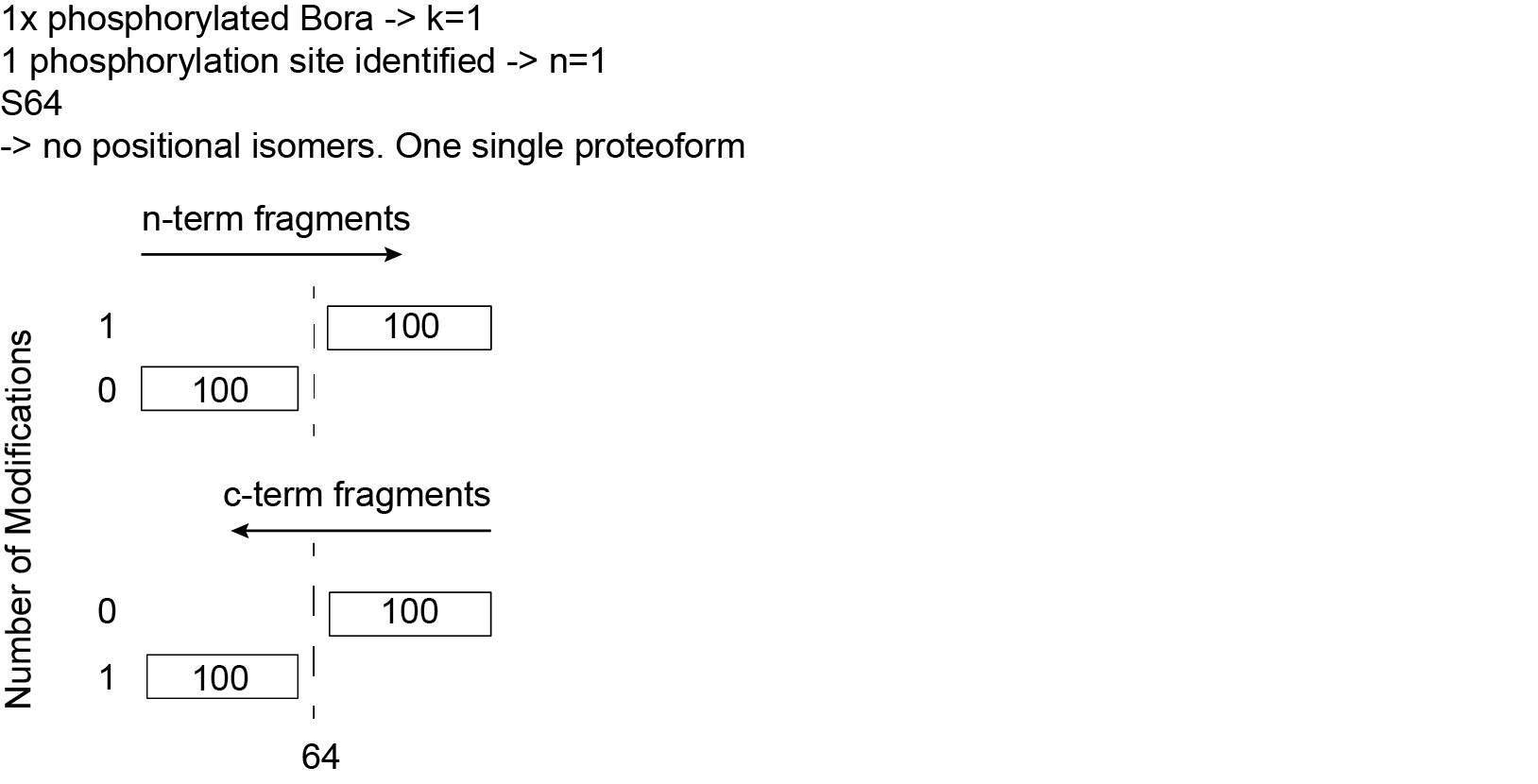


**Supplementary Figure 7 – Identification and quantification of singly phosphorylated Bora proteoforms.** Schematic depiction of the fragment ion ratios (in %) of singly phosphorylated Bora. One single phosphorylation site was identified at S64, resulting in one single proteoform.


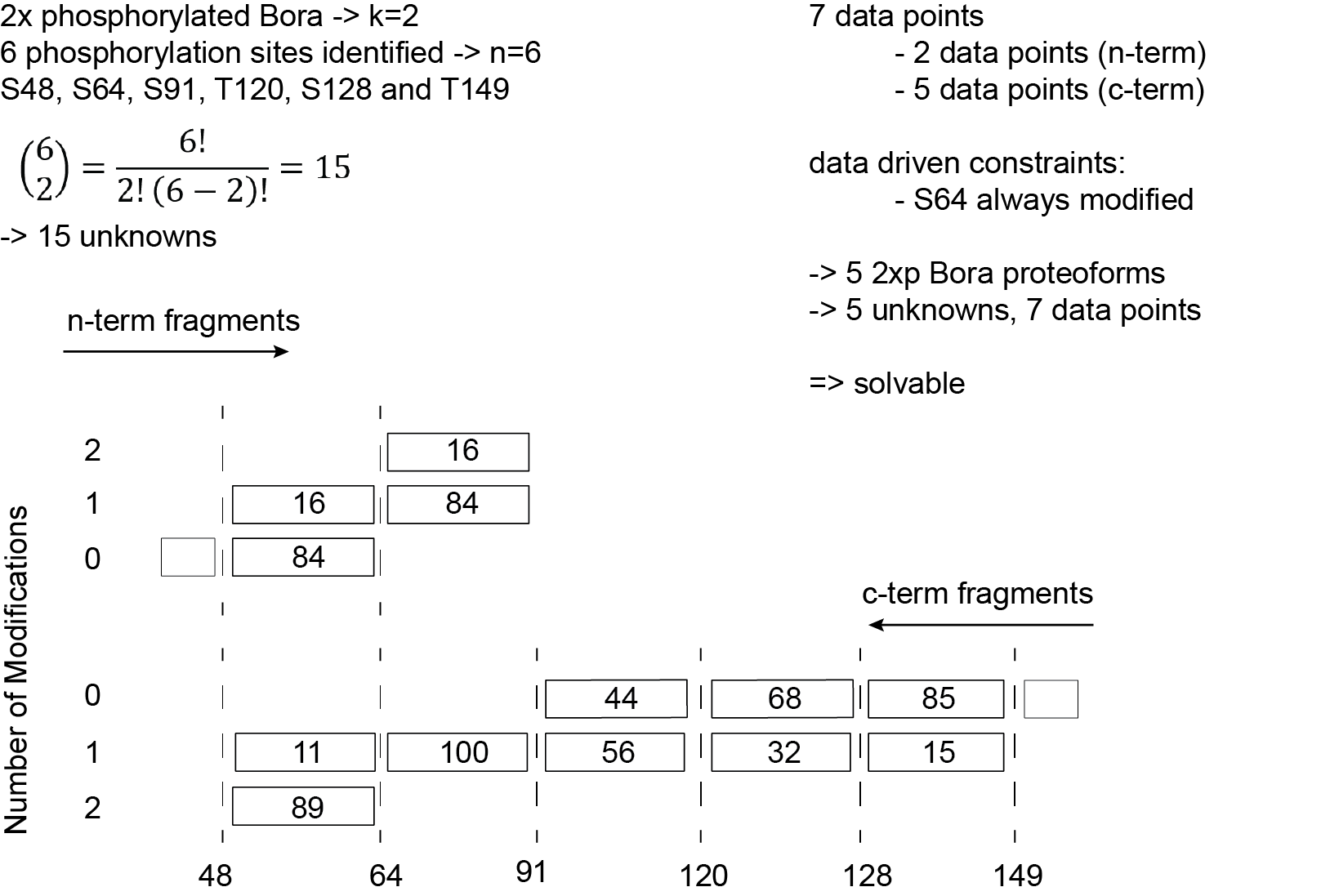


**Supplementary Figure 8 – Identification and quantification of doubly phosphorylated Bora proteoforms. Schematic depiction of the fragment ion ratios (in %) of doubly phosphorylated Bora.** 6 phosphorylation sites were identified at S48, S64, S91, T120, S128 and T149. Considering the equations above, the combination of these results in 15 theoretically possible proteoforms representing 15 unknowns. The 7 data points achieved experimentally do not allow proteoform quantification. The data shows that S64 is always phosphorylated. Applying this constraint reduces the number of unknowns to 5 potential proteoforms, which can be quantified with the 7 experimentally determined data points.


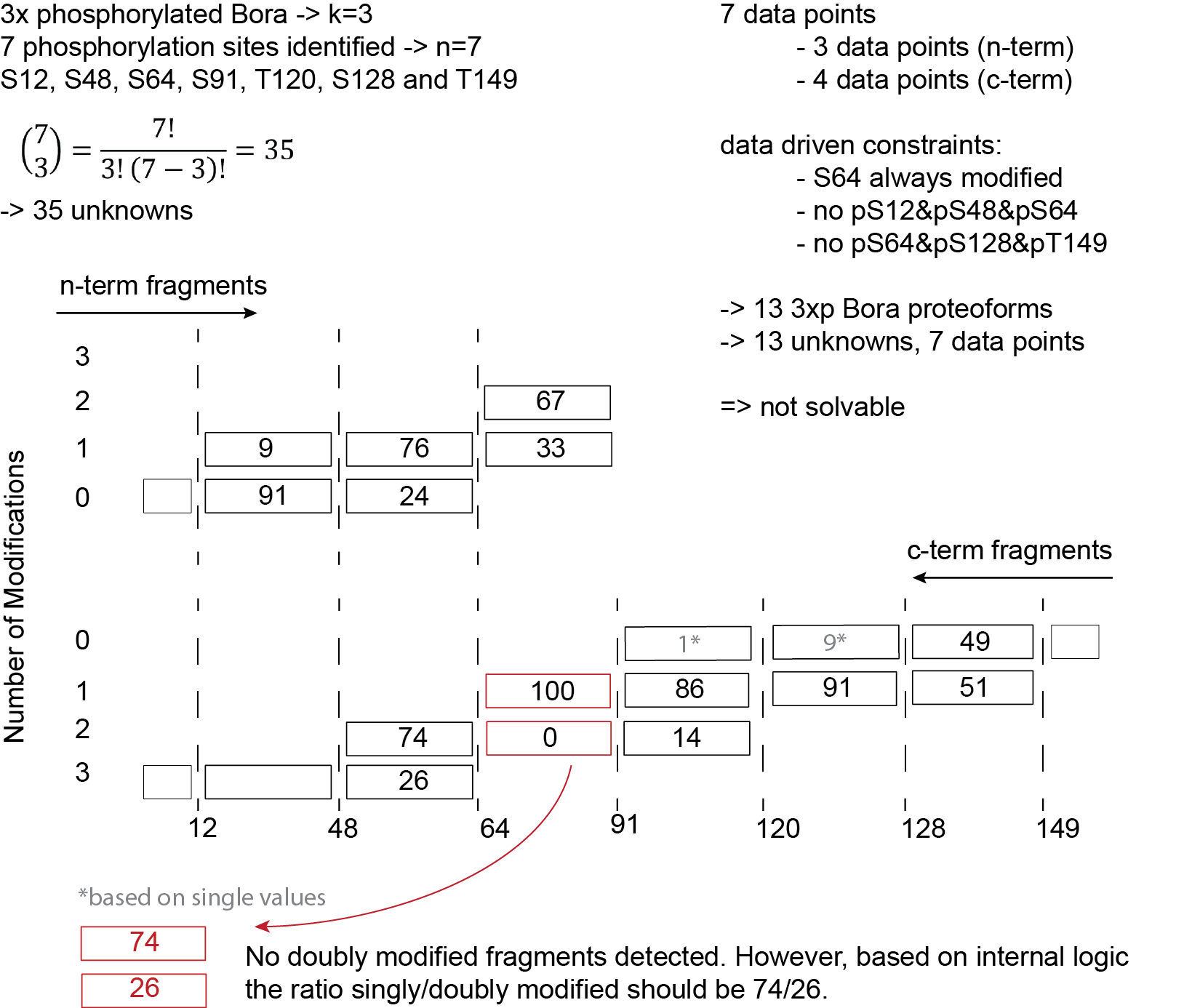


**Supplementary Figure 9 – Identification and quantification of triply phosphorylated Bora proteoforms.** Schematic depiction of the fragment ion ratios (in %) of triply phosphorylated Bora. 7 phosphorylation sites were identified at S12, S48, S64, S91, T120, S128 and T149. The combination of these results in 35 theoretically possible proteoforms, i.e. 35 unknowns. The 7 data points achieved experimentally do not allow proteoform quantification. The data show that S64 is always phosphorylated, and that phosphorylation at S12 and S48, and S128 and T149 are mutually exclusive. Applying these constraints leads to 13 potential proteoforms, which cannot be quantified with the 7 experimentally determined data points. However, they could be quantified if all 14 theoretically possible data points had been achieved. The ratios in grey are based on single values, so that no t-testing was possible and the presence of T120 has to be inferred from the 2x phosphorylated proteoforms. The ratios encased in red are the experimentally determined. However the presence of triply phosphorylated c-terminal fragments after S64 indicate, that doubly phosphorylated fragments must be present after S91. We therefore infer that 26% of the fragments are doubly phosphorylated in the stretch 64 to 91.

**References**

1. Schmidlin, T., Debets, D. O., van Gelder, C. A. G. H., Stecker, K. E., Rontogianni, S., van den Eshof, B. L., Kamper, K., Lips, E. H., van den Biggelaar, M., Peeper, D. S., Heck, A. J. R., and Altelaar, A. F. M. (2019) High-Throughput Assessment of Kinome-wide Activation States. *Cell Syst.* 9, 366–374
