# Supplementary material for: Quantifying Positional Isomers (QPI) by Top-Down Mass Spectrometry
Source: Mol Cell Proteomics. 2021 Mar 10;20:100070. doi: 10.1016/j.mcpro.2021.100070 (PMC8099777; doi:10.1016/j.mcpro.2021.100070)
Supplement: supplementary material [file mmc2.zip › 20181212_QPI_Manual.docx]

**Quantifying Positional Isomers (QPI) by top-down mass spectrometry**

Software manual v 1.0

12/12/2018


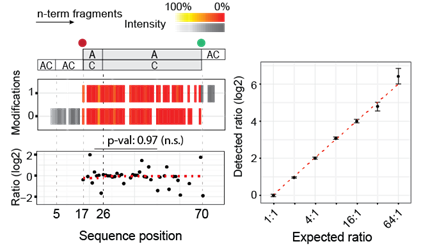


Contents

[Introduction 3](#_Toc532669239)

[Pre-requisites 3](#_Toc532669240)

[Data availability 3](#_Toc532669241)

[Quick start 4](#_Toc532669242)

[Quantifying positional isomers 6](#_Toc532669243)

# Introduction

QPI is implemented in the tool Topdown lab.

## Pre-requisites

The software runs on normal desktop pc’s with the following minimum specifications

1. Windows 7 or greater (64 bit)
2. 4gB internal memory
3. .NET 4.7 or greater

## Data availability

All raw-data, extracted peak lists and project files can be downloaded from

# Quick start

In this quick start, we focus on singly phosphorylated Bora (folder XXX). Before we assemble the datasets into the application, we need to fill in a couple of pieces of information, which need to be filled into the user interface:

**M/z accuracy:** 2 ppm

**Modification:** Phospho (STY)

**Number of modifications:** 1

**Minimum number of replicates:** 2 out of 3

**Fragmentation technique:** ETD

**Protein sequence:**

GAASMMGDVKESKMQITPETPGRIPVLNPFESPSDYSNLHEQTLASPSVFKSTKLPTPGKFRWSIDQLAVINPVEIDPEDIHRQALYLSHSRIDKDVEDKRQKAIEEFFTKDVIVPSPWTDHEGKQLSQCHSSKCTNINSDSPVGKKLTIHSEKSD

To launch the software, double-click on ‘TopdownLab.exe’ in the install folder (left panel); upon start the application will show up as follows (right panel).


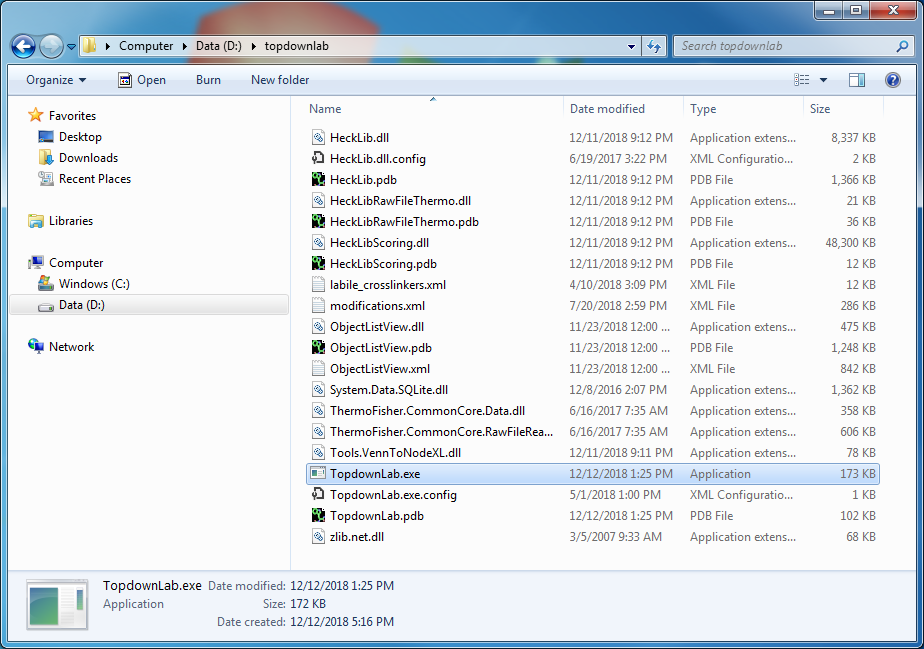

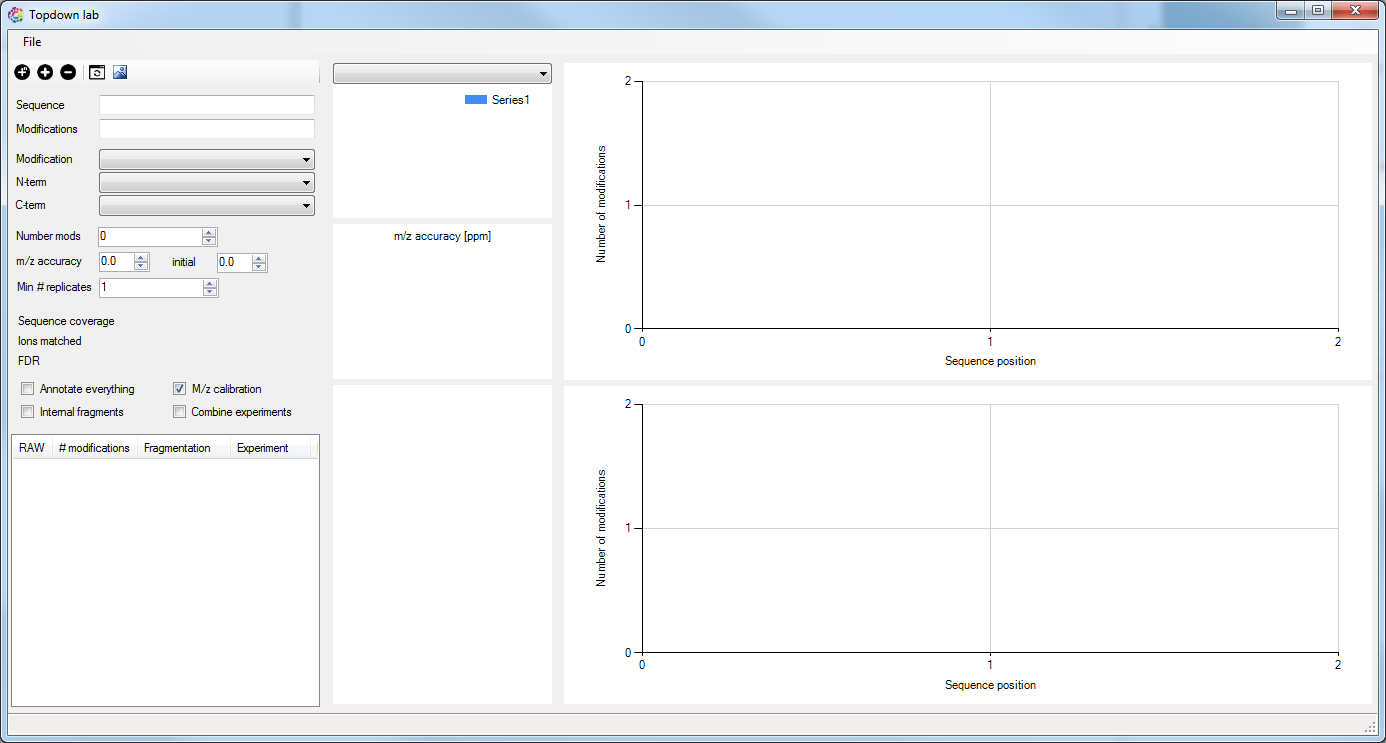


From here on it is possible to load data. Take the following steps:

1. Copy / fill in the information above into the user-interface.
2. Load the files with the option ‘Load multiple files’


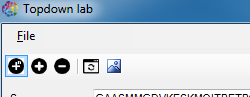


Select the files

- 20141202_AMB_pBora_AurA_10x_40MeOH_1FA_OT_60k_10uscans_924_ETD_6ms_19precZ-sc1.xls
- 20141202_AMB_pBora_AurA_10x_40MeOH_1FA_OT_60k_10uscans_924_ETD_6ms_19precZ-sc2.xls
- 20141202_AMB_pBora_AurA_10x_40MeOH_1FA_OT_60k_10uscans_924_ETD_6ms_19precZ-sc3.xls

And click Ok.

1. This list with files has now been updated with the three files. The fragmentation method for each of the files has however automatically been set to HCD, which needs to be changed to the correct method ETD. Double-click on the label HCD to pull up a pulldown list from which the appropriate method can be selected.


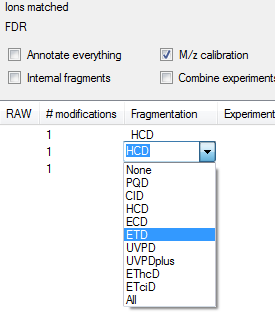


1. After updating, the update button needs to be clicked to start the analysis and update the view.


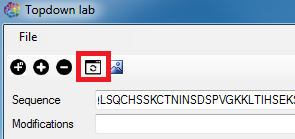


After these steps, the application shows the following. From the visualization, it is clear that with ETD the protein can be fragmented roughly halfway into the sequence, resulting into high sequence coverage as we see fragments originating from both termini. Furthermore, we can see that the phosphorylation group is located on position 64 from the behavior in the heatmaps.


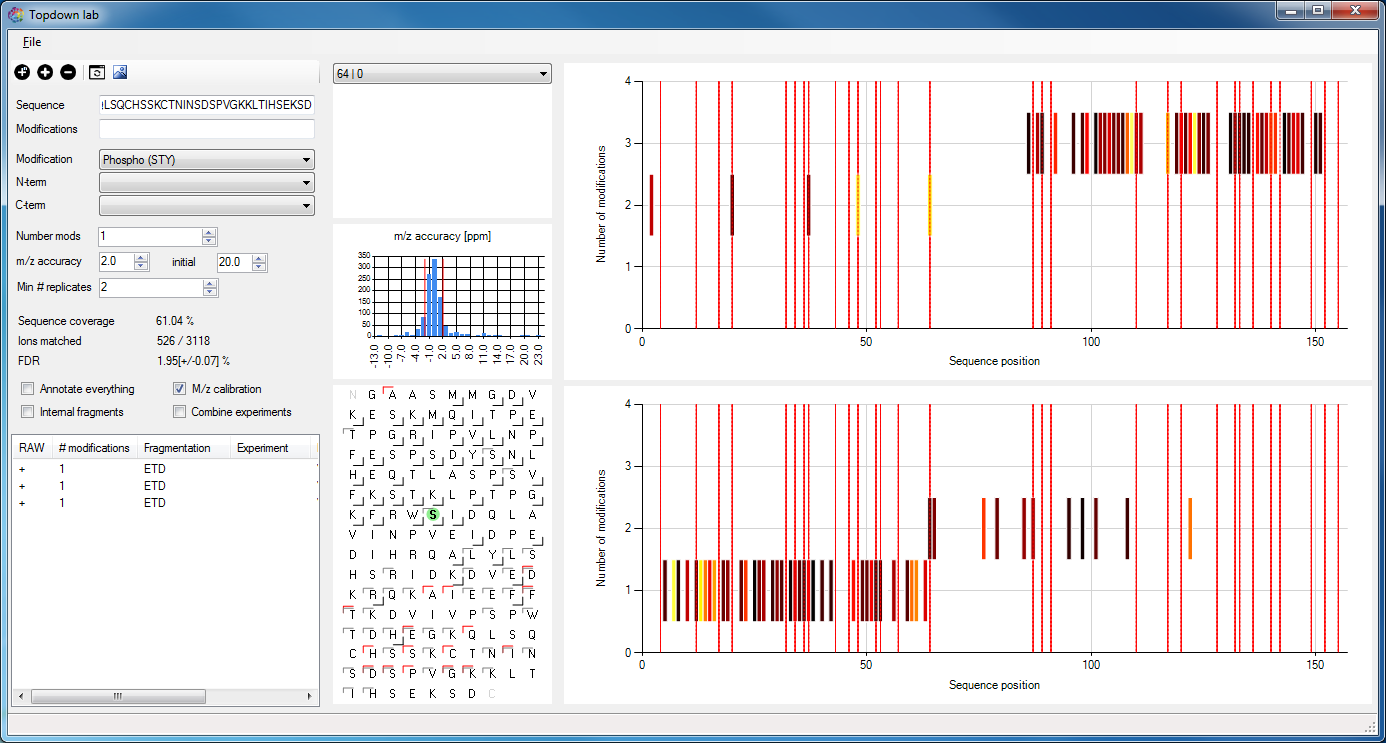


At any given point, all information can be stored in a project file (extension: .tdproject). For this select in the filemenu “File -> Save…”. The stored file can be loaded directly in the application (“File -> Open…”) which will restore all information filled into the user interface.

The software automatically performs many steps to make the assignment of the fragments at low FDR’s (in this case 1.95 +/-0.07 %; for over 500 peaks this means 10 are likely mis-assigned). For this purpose, it performs mass calibration of the individual spectra prior to combination. The mass accuracy is limited by the user to 2 ppm for assigning the peaks. The FDR itself is automatically calculated by 200 times scrambling the sequence and assigning theoretical fragments. The median number of matches +/- the standard deviation is reported.

# Quantifying positional isomers

To show how quantification of positional isomers works, we revert to a simpler system. For this purpose, we have selected Ubiquitin modified with a heavy Valine. The relevant information is listed below:

**M/z accuracy:** 1.9 ppm

**Modification:** Val6

**Number of modifications:** 1

**Minimum number of replicates:** 2 out of 3

**Fragmentation technique:** ETD

**Protein sequence:**

LQIFVKTLTGKTITLEVEPSDTIENVKAKIQDKEGIPPDQQRLIFAGKQLEDGRTLSDYNIQKESTLHLVLRLRGG

Select the files

- 20160502_AMB_labeledUb_MA1to3_1to1_2uM_50MeOH_1FA_2kV_2mTor_MS2_prec788_OT_240k_ETD6_2e5_100usc_1.csv
- 20160502_AMB_labeledUb_MA1to3_1to1_2uM_50MeOH_1FA_2kV_2mTor_MS2_prec788_OT_240k_ETD6_2e5_100usc_2.csv
- 20160502_AMB_labeledUb_MA1to3_1to1_2uM_50MeOH_1FA_2kV_2mTor_MS2_prec788_OT_240k_ETD6_2e5_100usc_3.csv

The rather short sequence of 77 amino acids allows for close to 100% sequence coverage. More importantly though, we observe stretches with fragments containing no and 1 modification (position 17 – 70).


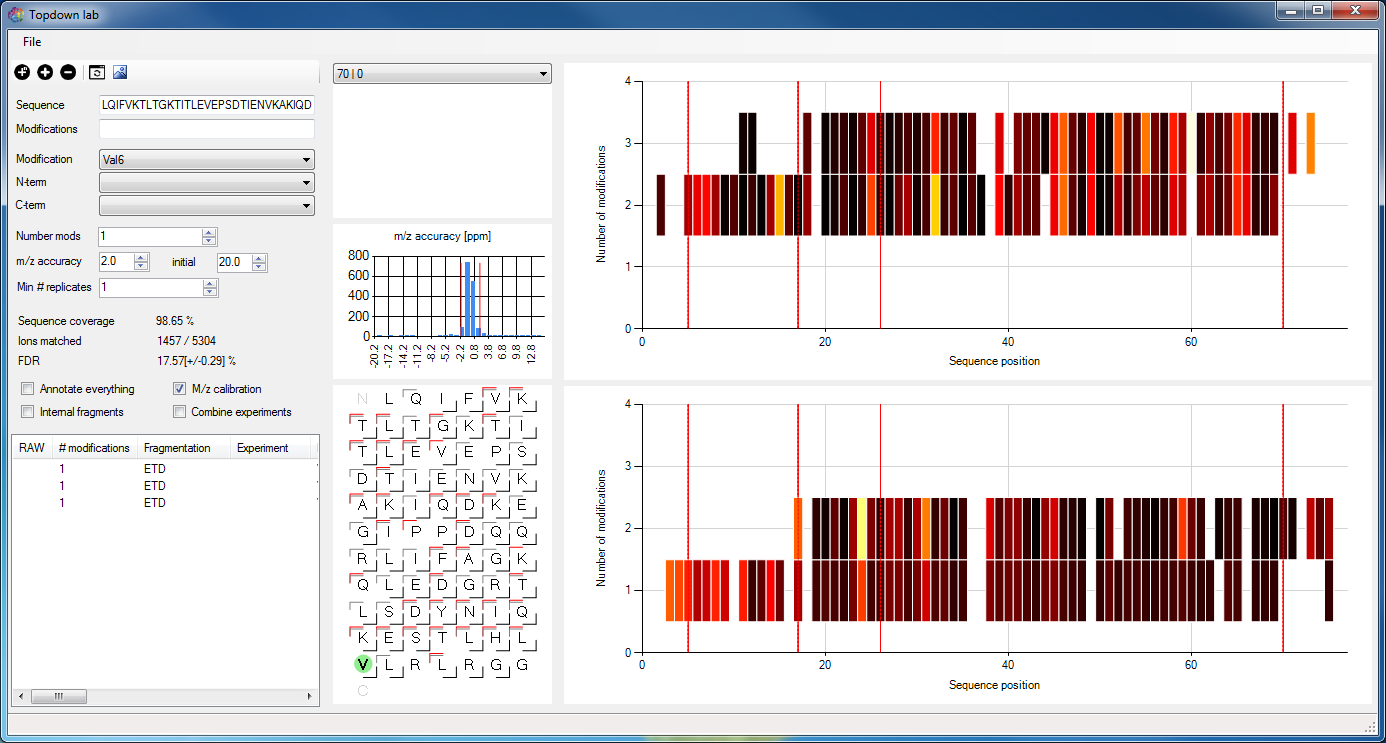


From the heatmap it looks like two positions in the c-terminal matches and 1 position in the n-terminal matches are mis-assigned. Given the rather high FDR of 17.57 +/- 0.29 % this is a likely explanation. To note, the FDR calculation is sensitive to the length of the sequence, which in this case is a little too short to provide 200x a completely random sequence – resulting in an artificially higher FDR rate. As the length gets longer this problem disappears and the FDR levels off to its true value.

This individual contribution of each of the positional isomers can be calculated from the ratio between no modification and 1 modification carrying fragments. To proceed with this, select ‘File -> Export -> Quant…’ and select a directory where multiple files can be exported. The resulting folder contains the following from which the individual contribution of the positional isomers can be determined.


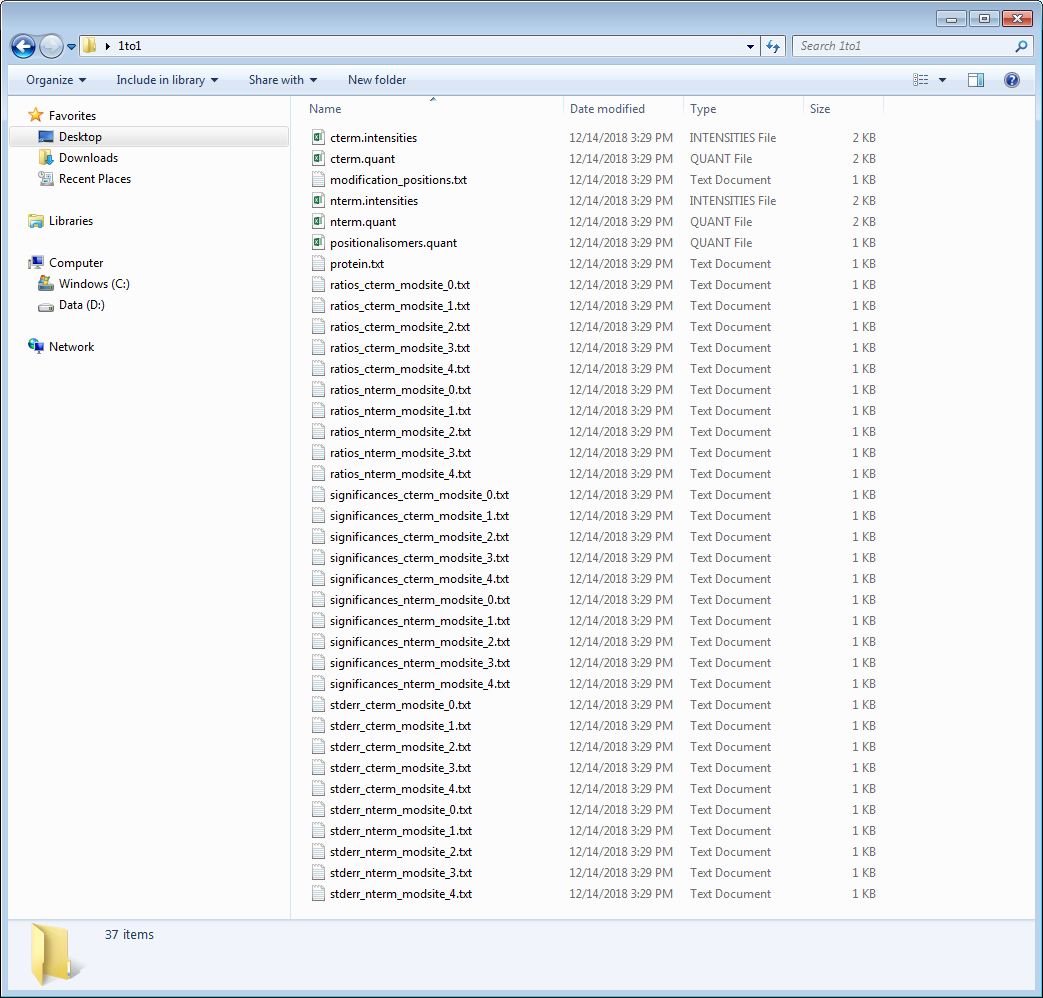


| **Filename** | **Description** |
| --- | --- |
| *.quant | The contribution of each ‘channel’ (i.e. 0 modifications, 1 modification, etc.) to the total abundance. These values are normalized and can be used to calculate the ratio within the stretch. |
| *.intensities | The raw intensities extracted from the peak-list and/or the raw-file. |
| ratios_*term_modsite_*.txt | The easiest location to read out the ratio between positional isomers. The files are exported for each modification site individually, and |
| stderror_*term_modsite_*.txt | The standard errors for the calculated ratios. |

The plot below has been built up out *.intensities (heatmap top-left panel), *.quant (point-plot bottom-left panel), and ratios_*term_modsite_*.txt + stderror_*term_modsite_*.txt for the line-plot (right panel)


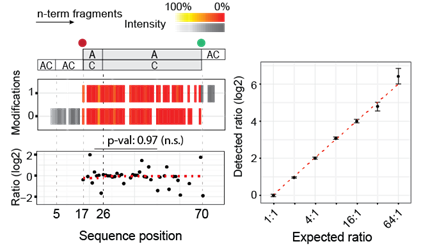


Upon analyzing all samples with ratios of 1:1 up to 64:1, built up out of steps of 2, we can see that the ratios conform to the expected trend (red dotted line).
